# The Isoform GC1f of the Vitamin D Binding Protein Is Associated with Bronchiectasis Severity

**DOI:** 10.3390/biomedicines9111573

**Published:** 2021-10-29

**Authors:** Martina Oriano, Stefano Aliberti, Franca Rosa Guerini, Cristina Agliardi, Carlotta Di Francesco, Alice Gelmini, Leonardo Terranova, Milena Zanzottera, Paola Marchisio, Mario Clerici, Francesco Blasi

**Affiliations:** 1 Department of Pathophysiology and Transplantation, University of Milan, 20122 Milan, Italy; carlotta.difrancesco@unimi.it (C.D.F.); alice.gelmini@unimi.it (A.G.); leonardo.terranova@unimi.it (L.T.); paola.marchiso@unimi.it (P.M.); francesco.blasi@unimi.it (F.B.); 2 Respiratory Unit and Adult Cystic Fibrosis Center, Internal Medicine Department, Fondazione IRCCS Ca’ Granda Ospedale Maggiore Policlinico, 20122 Milan, Italy; 3 Department of Biomedical Sciences, Humanitas University, Via Rita Levi Montalcini 4, 20072 Pieve Emanuele, Italy; 4 Respiratory Unit, IRCCS Humanitas Research Hospital, Via Manzoni 56, 20089 Rozzano, Italy; 5 IRCCS Fondazione Don Carlo Gnocchi ONLUS, 20148 Milan, Italy; cagliardi@dongnocchi.it (C.A.); mzanzottera@dongnocchi.it (M.Z.); 6 Paediatric Highly Intensive Care Unit, Fondazione IRCCS Ca’ Granda Ospedale Maggiore Policlinico, 20122 Milan, Italy

**Keywords:** bronchiectasis, vitamin D, DBP, rehabilitation

## Abstract

Vitamin D modulates immune responses and its deficiency has been observed in more than 60% of bronchiectasis patients. Vitamin D binding protein (DBP) is coded by the GC gene, is involved in the transport of vitamin D, and includes a number of isoforms based on single nucleotide polymorphisms (SNPs) in the coding region at rs7041 and rs4855. We evaluated the possible clinical impact of DBP polymorphisms and isoforms in an observational, cross-sectional study conducted in 116 bronchiectasis patients, who were genetically characterized for rs4588 and rs7041 SNPs. Results showed that the GC1f isoform (rs7041/rs4588 A/G) correlated with a more severe disease (18.9% vs. 6.3%, *p* = 0.038), a higher incidence of chronic infections (63.6% vs. 42%, *p* = 0.041), and a lower BACI score (0.0 (0.0, 2.5) vs. 3.0 (0.0, 3.0), *p* = 0.035). Moreover, blood concentration of vitamin D was higher in patients carrying GC1s (median (IQR): 20.5 (14.3, 29.7 vs. 15.8 (7.6, 22.4), *p* = 0.037)). Patients carrying GC1f isoform have a more severe disease, more chronic infections and lower asthmatic comorbidity in comparison to those without the GC1f isoform. Presence of the GC1s isoform (rs7041/rs4588 C/G) seems to be associated to a milder clinical phenotype with increased vitamin D levels and lower comorbidities score.

## 1. Introduction

Bronchiectasis is a chronic respiratory disease characterized by abnormal dilation of bronchi with patients experiencing daily cough, sputum production and frequent exacerbations [1]. Pulmonary infections often become chronic leading to a vicious circle of airway inflammation [2]. Host–pathogen interaction is, nowadays, a hot topic in bronchiectasis investigations, and the identification of treatable traits is one of the main interests of both scientists and clinicians [3].

Among candidate treatable traits in bronchiectasis, vitamin D and its pathway seem to be among the most promising. Vitamin D is involved in pulmonary immunity and its deficiency has been observed in more than 60% of bronchiectasis patients [4,5] and correlates to disease and radiological severity, increased airway inflammation and poor quality of life [4,5]. Vitamin D binding protein (DBP) is involved in the transport of vitamin D, expressed in different tissues, and produced also by neutrophils. Other functions of this protein include macrophage activation after conversion to macrophage-activating factor (DBP-MAF) by enzymes released from lymphocytes, modulation of neutrophils and monocytes chemotaxis through the increased production of C5-derived peptides and actin scavenging [6,7,8,9].

DBP is coded by the single copy *GC* gene (NCBI GENE ID2638) [6]. A great number of genetic variants have been identified both in the intronic and exonic portion of this gene. Isoforms of this protein were isolated through functional studies, and now we know that they are based on single nucleotide polymorphisms (SNPs) in the coding region at rs4855 and rs7041. SNPs and isoforms of DBP have been associated with asthma and COPD [6,10,11,12]. No data are available to date in bronchiectasis. Vitamin D regulates more than 900 genes and it is involved in both innate and adaptive immunity. Polymorphisms in the *GC* gene can be involved in both vitamin D bioavailability and have also direct effects on immunity in lungs and, therefore, on the patient’s disease state [13,14].

For these reasons, the aim of this work was to evaluate the impact of DBP polymorphisms and isoforms on bronchiectasis patients’ characteristics.

## 2. Materials and Methods

### 2.1. Study Design and Population

This observational, cross-sectional study enrolled consecutive adults (aged ≥18 years) with bronchiectasis referring to the Bronchiectasis Program of the Fondazione IRCCS Ca’ Granda Ospedale Maggiore Policlinico, Milan, Italy, between March 2017 and March 2019. Patients with clinically (daily sputum production) and radiologically significant bronchiectasis (at least one lobe involvement on chest CT) enrolled during clinical stability (at least 1 month apart from the last exacerbation and antibiotic course). Patients with cystic fibrosis or traction bronchiectasis due to pulmonary fibrosis were excluded along with patients under supplementation with vitamin D. Informed consent was obtained from all the subjects prior to inclusion in the study. The study was conducted according to the guidelines of the Declaration of Helsinki and was approved by the institutional review board of Institute (Comitato Etico Milano Area 2, #255_2020, 8 April 2020)

### 2.2. Study Procedures

Peripheral blood samples were collected from all bronchiectasis patients. Patients underwent clinical, radiological, microbiological, quality of life and functional evaluation during routine visits. We carried out 25-hydroxy vitamin D blood test, immunological functionality and white blood cells count as per clinical practice.

### 2.3. GC Single Nucleotide Polymorphisms (SNPs) Genotyping

GC SNPs (rs4588 and rs7041) were evaluated by allelic discrimination real-time PCR using pre-designed TaqMan^®^ probes (C___8278879_10, and C___3133594_30 assays respectively, ThermoFisher Scientific, Waltham, MA, USA) on DNA extracted from peripheral blood using the QIAsymphony platform as per the manufacturer’s instructions. The PCR consisted of a hot start at 95 °C for 10 min followed by 40 cycles of 94 °C for 15 s and 60 °C for 1 min. Fluorescence detection (VIC and FAM fluorophores) takes place at a temperature of 60 °C. All assays were performed in 10 µL reactions, using TaqMan Genotyping Master Mix and 20 ng of DNA on 96-well plates using a CFX96 instrument (Bio-Rad, Hercules, CA, USA). Control samples representing all possible genotypes and a negative control were included in each reaction [15].

Polymorphisms were considered throughout the manuscript as: SNPs genotype, allele, isoform and isoforms’ phenotype. Isoforms genotype in the two loci are provided in Table 1.

### 2.4. Clinical Evaluation

Disease severity was assessed through both Bronchiectasis Severity Index (BSI) and FACED score (evaluating FEV_1_, Age, Chronic infection with Pseudomonas, Radiological Extension and Dyspnea) [16,17]. A modified Reiff score was used to assess radiological severity of bronchiectasis. It rates the number of involved lobes (with the lingula considered to be a separate lobe) and the degree of dilatation (range: 1–18) [18]. All bacteriology was performed on spontaneous sputum samples as previously described [19]. Murray-Washington criteria for sputum quality were used in all cases, with all samples having less than 10 squamous cells and more than 25 leukocytes per low-power microscope field. Chronic infection is defined as 2 isolation of the same bacteria at least 3 months apart over 12 period [20]. Quality of life was assessed through the Quality of life -Bronchiectasis (QoL-B) questionnaire [21]. Asthma was diagnosed according to the latest international guidelines (Global Initiative for Asthma. Pocket Guide for Asthma Management and Prevention. Available online: https://ginasthma.org/pocket-guide-for-asthma-management-and-prevention accessed on 2 April 2021).

### 2.5. Statistical Analysis

Variables were collected in an ad hoc electronic form. Qualitative variables were summarized with absolute and relative (percentage) frequencies, whereas quantitative variables with medians (interquartile ranges, IQR). Differences between groups were statistically assessed with chi-squared or Fisher exact tests when appropriate for qualitative variables, whereas with Mann–Whitney tests for quantitative non-parametric variables. A two-tailed *p*-value was considered statistically significant when less than 0.05. Analyses were performed considering genotype, allele, isoform and isoform phenotype for the considered SNPs. The statistical software SPSS version 25 (IBM, Armonk, NY, USA) was used for all statistical computations.

## 3. Results

We included 116 bronchiectasis patients (78 (67.2%) female, age median (IQR) 62.0 (48.8, 72.0)) in the study. Vitamin D concentration in blood was median (IQR) 20.4 (13.0, 28.4) ng/mL. A full description of clinical characteristics of the study population is reported in Table S1 of supplementary file.

Frequency of DBP isoforms and isoform phenotypes is reported in Table 2. GC1s isoform was found in 95 (81.9%) of the enrolled patients, GC2 in 55 (47.4%) and GC1f in 37 (31.9%).

Full description of rs7041 and rs4588 alleles and genotypes are reported in Table S2 of supplementary file.

### 3.1. GC Isoforms and Clinical Characteristics

#### 3.1.1. GC1f Isoform

A significant difference in disease severity measured with FACED score was found between GC1f and the other isoforms (FACED severe, *n* (%): GC1f 7 (18.9%) vs. other isoforms 5 (6.3%), *p* = 0.038).

Chronic infection was higher in patients with GC1f isoform 12 (63.6%) compared to the others 29 (42%), (*p* = 0.041), although no differences in terms of chronic infection by specific bacteria were found in the study groups.

Patients with GC1f isoform showed higher values at QoL-B emotional section [median (IQR): GC1f 83.3 (75–100) compared to the other isoforms 75 (50–89.6), (*p* = 0.019), along with a lower asthma prevalence as comorbidity [GC1f 2 (5.4%) vs. other isoforms 18 (22.8%), *p* = 0.032].

Full comparison of the study groups is reported in Table 3.

#### 3.1.2. GC1s Isoform

Patients with GC1s were similar to the other isoforms in all clinical characteristics but vitamin D peripheric blood levels were higher in the group carrying this isoform GC1s 20.5 (14.3, 29.7) vs. others 15.8 (7.6, 22.4), (*p* = 0.037). Moreover, BACI score was significantly lower in GC1s: 0.0 (0.0, 2.5) vs. other isoforms: 3.0 (0.0, 3.0), (*p* = 0.035), although no difference in the comorbidities that may be associated to vitamin D deficiency was found (Table 4).

#### 3.1.3. GC2 Isoform

Patients with GC2 showed a lower rate of hospitalization in the year prior enrolment (GC2 2 (3.6%) vs. other isoforms 11 (18.0%), *p* = 0.015) along with a decreased incidence of osteoporosis (GC2 1 (1.8%) vs. other isoforms 7 (11.5%), *p* = 0.04) (Table S3 of the supplementary file).

### 3.2. GC Isoform Phenotypes and Clinical Characteristics

Hospitalization rate was increased in patients with GC1s-GC1f and GC2-GC2 in comparison to GC1f-GC2 (GC1s-GC2 0 (0.0%) vs. GC2-GC2 2 (25.0%), *p* = 0.003; GC1s-GC1f 7 (29.2%) vs. GC1s-GC2 0 (0.0%), *p* = 0.001). Moreover, asthmatic comorbidity was less frequent in patients with GC1s-GC1f 1(4.2%) vs. GC2-GC2 4 (50.0%), (*p* = 0.002). Full comparison of the study groups is reported in Table S4 of supplementary file.

## 4. Discussion

The most important finding of the present study is that bronchiectasis patients with the GC1f isoform have a more severe disease, more frequency of chronic infections and lower asthmatic comorbidity in comparison to those without the GC1f isoform. Moreover, the GC1s isoform seems to be associated to a milder phenotype with increased vitamin D levels and lower comorbidities score (BACI).

Notably, the link between GC1f isoform and clinical/biological data is mainly based on observational data and correlations, not proving any mechanistic rationale.

The effect of *GC* isoforms on chronic respiratory diseases is various. Studies on asthmatic patients reported GC2 isoform to be associated with asthma susceptibility and to an allergic-like immune response [12,22]. Experiences in COPD reported a decreased disease risk with the GC2 variant, whereas the same isoform was associated with increased risk of bronchiectasis in these patients and SNPs in rs7041 were related to a1-antitrypsin deficiency. The isoform GC1f was reported instead as risk factor in this respiratory disease [22].

Vitamin D in our cohort resulted in being 20.5 ng/mL (median level), higher than in other bronchiectasis patients reported by Chalmers and colleagues [4] (24.7 nmol/L, approximately 9.9 ng/mL) and slightly higher than that presented by Ferri et al., with 17.3 ng/mL [5]. We can speculate that the difference between our cohort and the Scottish one should be caused by a diversity in sun exposure of the two countries. We found an increase of vitamin D levels in patients with GC1s isoform compared to those without this isoform. Data in literature reporting vitamin D serum levels in association with GC isoforms are contradictory. The isoform associated with the lowest levels of vitamin D is GC2, and GC2-GC2 phenotype; however, GC1s is usually associated with an intermediate value of vitamin D [23]. The molecular mechanism underneath the association of low vitamin D blood levels are still unknown [23].

GC1f prevalence seemed related to a severer clinical profile in the disease cohort. Bronchiectasis is a chronic respiratory disease that was associated with chronic infection and inflammation in lungs. Among all the genes activated by the vitamin D pathway, we can find some involved in both innate and adaptative immunity [22]. DBP itself seems to be involved in C5 modulation that leads a modification in neutrophils and monocytes chemotaxis [6]. The association of vitamin D pathway and DBP with immunity reported in literature should explain some of our findings, even if further studies should be needed in order to understand the effect of SNPs in *GC* in relation to local inflammation and infection in lungs.

The radiological severity evaluated through the Reiff score was not associated with the GC1f isoform. This is not surprising in view of the fact that the Reiff score was not originally derived in a population of non-cystic fibrosis bronchiectasis and because it might not capture the complexity of the radiological manifestations of the disease (e.g., bronchial wall thickness, sputum plug, etc.). As a consequence of this, in clinical practice discrepancies are often observed between the radiological and the clinical severity due to the presence of several confounders and it being a very complex disease.

Furthermore, it seems that the presence of isoform GC1f does not affect the number of exacerbations, disease duration, or patient’s BMI. Thus the presence and absence of the isoform GC1f might not be closely related to disease progression.

Poor data in literature associated GC1s with respiratory diseases. GC1s-GC1s have been associated in literature with high levels of DBP in sputum of COPD patients and with higher pulmonary obstruction in this disease [24]. Another study reported GC1s variant significantly more frequent in non-smoker controls. Researchers speculated that GC1s may have a role in the detoxification of substances found in smoke [25].

Both rs4588 and rs7041, polymorphisms responsible for the three isoforms, are located in the exon 11 of the *GC* gene. [6]. These modifications in the amino acid conformation of the protein should lead to functional difference including half-life, affinity to the substrate, cell transit time and others that the scientific community has not fully disclosed yet [6].

These functional differences among isoforms may be involved in the associations we found with clinical data in bronchiectasis.

### Strength and Limitations

This study has some limitations. Firstly, this is a monocentric study reporting data from Italy, a south-European country that has a natural higher exposure to sunlight, as a consequence, a bias in vitamin D levels evaluation should have been introduced and this could be one of the reasons explaining why we did not find differences in vitamin D levels among isoforms. Secondly, data regarding reversibility testing, asthma treatments, or pack/years history were not collected, and clinical data on exacerbation rate, antibiotic use, and BMI were missing. All this information would have allowed us to define the role of GC1f across different subpopulations of bronchiectasis patients more precisely.

All these caveats notwithstanding, the study has some potentially interesting clinical implications. Bronchiectasis has a large heterogeneity in symptoms, aetiology, disease severity and biological characteristics, and the identification of a new biomarker could help in the stratification of patients and might contribute to the development of a personalized approach to the disease.

As mentioned above, further studies investigating inflammation and bacterial colonization of lungs, as well as cytological assessment and DBP quantification in association with polymorphisms in the *GC* gene, should be carried out in the future in order to develop a deeper knowledge of the clinical associations of these genetic factors to bronchiectasis. Moreover, these data on clinical associations in the bronchiectasis cohort should be confirmed in a larger and more generalizable population.

### 5. Conclusions

In conclusion, we demonstrated an increased disease severity, increased chronic infection in adult patients with the GC1f isoform of DBP, along with an association of the GC1s isoform with a milder phenotype with increased vitamin D levels and a lower comorbidities score. These results may represent a step towards the identification of a new biomarker that should help in the stratification of patients and may contribute to the development of a personalized approach to bronchiectasis treatment.

## Figures and Tables

**Table 1:** Isoform genotype in rs7041 and rs4588.

Isoform	*GC* rs7041	*GC* rs4588
GC1f	A	G
GC1s	C	G
GC2	A	T

**Table 2:** Isoforms of vitamin D binding protein (DBP) in bronchiectasis patients.

Isoform	Genotype (rs7041/rs4588)	Bronchiectasis *n* (%)
GC1s	C/G	95 (81.9)%
GC1f	A/G	37 (31.9)%
GC2	A/T	55 (47.4)%
**Isoform Phenotype**	**Genotype (rs7041/rs4588)**	**Bronchiectasis *n* (%)**
GC1f-GC1f	A/G-A/G	0 (0)%
GC1s-GC1f	C/G-A/G	24 (20.7)%
GC1s-GC1s	C/G-C/G	37 (31.9)%
GC1s-GC2	C/G-A/T	34 (29.3)%
GC1f-GC2	A/G-A/T	13 (11.2)%
GC2-GC2	A/T-A/T	8 (6.9)%

**Table 3:** Comparison of clinical characteristics of patients with GC1f isoform vs. others.

Demography				
		GC1f (*N* = 37)	Other Isoforms (*N* = 79)	*p*-Value
Sex (Female)		26 (70.3%)	52 (65.8%)	0.634
Age		61.0 (53.0, 71.0)	63.0 (48.0, 72.0)	0.528
BMI		22.0 (19.0, 25.0)	21.7 (19.0, 24.2)	0.902
**Radiology**				
Reiff score		4.0 (3.0, 6.0)	4.0 (3.0, 4.5)	0.615
**Disease Severity**				
BSI score		7.0 (4.0, 10.0)	6.0 (3.5, 9.0)	0.318
BSI risk classes	mild	11 (29.7%)	29 (36.7%)	0.675
	moderate	12 (32.4%)	26 (32.9%)	
	severe	14 (37.8%)	24 (30.4%)	
BSI Moderate-severe		26 (70.3%)	50 (63.3%)	0.461
BSI Severe		14 (37.8%)	24 (30.4%)	0.425
FACED score		2.0 (1.0, 4.0)	2.0 (1.0, 4.0)	0.669
FACED risk classes	mild	21 (56.8%)	42 (53.2%)	0.055
	moderate	9 (24.3%)	32 (40.5%)	
	severe	7 (18.9%)	5 (6.3%)	
FACED Moderate–severe		16 (43.2%)	37 (46.8%)	0.717
FACED Severe		7 (18.9%)	5 (6.3%)	0.038
**Clinical Status**				
Exacerbation in the previous year		2.0 (1.0, 3.0)	2.0 (1.0, 3.0)	0.887
Hospitalization (at least one previous year)		7 (19.4%)	6 (7.6%)	0.063
**Comorbidities**				
BACI		0.0 (0.0, 3.0)	0.0 (0.0, 3.0)	0.505
Osteoporosis		2 (5.4%)	6 (7.6%)	0.664
Depression		2 (5.4%)	10 (12.7%)	0.232
Anxiety		1 (2.7%)	4 (5.1%)	0.56
Asthma		2 (5.4%)	18 (22.8%)	0.021
**Lung Function**				
FEV1%		79.5 (69.8, 102.0)	84.5 (69.2, 98.5)	0.602
FEV1% < 50%		6 (16.7%)	9 (11.5%)	0.451
FEV1% < 35%		3 (8.3%)	5 (6.4%)	0.709
**Standard Microbiology**				
Chronic infection		21 (57.6%)	29 (36.7%)	0.041
Chronic *P. aeruginosa*		12 (36.4%)	18 (26.1%)	0.287
Chronic *H. influenzae*		2 (6.1%)	4 (5.8%)	0.958
Chronic *MSSA*		4 (12.1%)	4 (5.8%)	0.266
Chronic *A. xylosoxidans*		1 (3.0%)	1 (1.4%)	0.59
Chronic Others		2 (2.9%)	2 (6.1%)	0.441
**Aetiology**				
Idiopathic		17 (45.9%)	46 (58.2%)	0.477
Primary Ciliary Dyskinesia		5 (13.5%)	5 (6.3%)	
Primary immunodeficiency		4 (10.8%)	9 (11.4%)	
Post Infective		3 (8.1%)	3 (3.8%)	
Secondary Immunodeficiency		4 (10.8%)	2 (2.5%)	
Others *		4 (10.8%)	14 (17.8%)	
**QoL-B Questionnaire**				
Physical section		66.7 (43.4, 86.7)	60.0 (41.3, 80.0)	0.459
Role section		70.0 (53.3, 93.3)	66.7 (49.2, 86.7)	0.502
Vitality section		61.2 (44.4, 77.8)	55.6 (33.3, 66.7)	0.232
Emotion section		83.3 (75.0, 100.0)	75.0 (50.0, 85.4)	0.019
Social section		75.0 (39.6, 91.7)	58.3 (41.7, 83.3)	0.452
Treatment Burden section		77.8 (58.4, 77.8)	66.7 (55.6, 77.8)	0.095
Health section		44.4 (33.3, 66.7)	37.5 (16.7, 58.3)	0.25
Respiration section		74.1 (66.7, 81.5)	74.1 (58.3, 81.5)	0.451
**Vitamin D**				
Vitamin D (ng/mL)		20.2 (11.0, 26.0)	20.4 (14.8, 29.1)	0.224

* Other includes COPD, Connective Tissue Diseases, Alpha1-antitrypsin deficiency, ABPA, Asthma, CFTR-RD, Aspiration. BSI: Bronchiectasis severity index; BACI: Bronchiectasis Aetiology and Co-Morbidity Index; FEV1: Forced expiratory volume first second.

**Table 4:** Comparison of clinical characteristics of patients with GC1s isoform vs. others.

Demography				
		GC1s (*N* = 95)	Other Isoforms (*N* = 21)	*p*-Value
Sex (Female)		64 (67.4%)	14 (66.7%)	0.951
Age		62.0 (48.0, 72.0)	62.0 (49.0, 71.0)	0.991
BMI		21.6 (19.0, 24.2)	22.0 (19.0, 26.0)	0.397
**Radiology**				
Reiff score		4.0 (3.0, 5.5)	4.0 (3.0, 6.0)	0.746
**Disease Severity**				
BSI score		6.0 (3.5, 9.0)	6.0 (4.0, 10.0)	0.793
BSI risk classes	mild	33 (34.7%)	7 (33.3%)	0.993
	moderate	31 (32.6%)	7 (33.3%)	
	severe	31 (32.6%)	7 (33.3%)	
BSI Moderate-severe		62 (65.3%)	14 (66.7%)	0.903
BSI Severe		31 (32.6%)	7 (33.3%)	0.951
FACED score		2.0 (1.0, 4.0)	3.0 (2.0, 4.0)	0.3
FACED risk classes	mild	53 (55.8%)	10 (47.6%)	0.722
	moderate	33 (34.7%)	8 (38.1%)	
	severe	9 (9.5%)	3 (14.3%)	
FACED Moderate-severe		42 (44.2%)	11 (52.4%)	0.496
FACED Severe		9 (9.5%)	3 (14.3%)	0.512
**Clinical Status**				
Exacerbation in the previous year		2.0 (1.0, 3.0)	2.0 (1.0, 3.0)	0.449
Hospitalization (at least one previous year)		11 (11.6%)	2 (10.0%)	0.839
**Comorbidities**				
BACI		0.0 (0.0, 2.5)	3.0 (0.0, 3.0)	0.035
Osteoporosis		8 (8.4%)	0 (0.0%)	0.168
Depression		12 (12.6%)	0 (0.0%)	0.085
Anxiety		90 (94.7%)	21 (100.0%)	0.282
Asthma		15 (15.8%)	5 (23.8%)	0.379
**Lung Function**				
FEV1%		84.0 (70.0, 101.0)	83.0 (56.0, 88.0)	0.233
FEV1% < 50%		11 (11.8%)	4 (19.0%)	0.377
FEV1% < 35%		5 (5.4%)	3 (14.3%)	0.149
**Standard Microbiology**				
Chronic infection		41 (48.2%)	9 (52.9%)	0.723
Chronic *P. aeruginosa*		24 (28.2%)	6 (35.3%)	0.56
Chronic *H. influenzae*		6 (7.1%)	0 (0.0%)	0.259
Chronic *MSSA*		8 (9.4%)	0 (0.0%)	0.188
Chronic *A. xylosoxidans*		1 (1.2%)	1 (5.9%)	0.201
Chronic Others		3 (3.5%)	1 (5.9%)	0.648
**Aetiology**				
Idiopathic		47 (49.5%)	16 (76.2%)	0.739
Primary Ciliary Dyskinesia		10 (10.5%)	0 (0.0%)	
Primary Immunodeficiency		9 (9.5%)	4 (19.0%)	
Post Infective		6 (6.3%)	0 (0.0%)	
Secondary Immunodeficiency		5 (5.3%)	1 (4.8%)	
Others *		18(18.9%)	0 (0%)	
**QoL-B Questionnaire**				
Physical section		63.4 (41.3, 80.0)	55.6 (40.0, 70.0)	0.608
Role section		66.7 (53.3, 86.7)	73.3 (56.6, 90.0)	0.873
Vitality section		55.6 (33.3, 66.7)	55.6 (44.4, 77.8)	0.724
Emotion section		75.0 (56.2, 91.7)	75.0 (62.5, 95.8)	0.684
Social section		58.3 (41.7, 83.3)	83.3 (62.5, 87.5)	0.147
Treatment Burden section		66.7 (55.6, 77.8)	66.7 (55.6, 77.8)	0.855
Health section		37.5 (22.9, 58.3)	44.4 (23.6, 58.4)	0.995
Respiration section		74.1 (58.5, 82.8)	74.1 (63.0, 77.8)	0.478
**Vitamin D**				
Vitamin D (ng/mL)		20.5 (14.3, 29.7)	15.8 (7.6, 22.4)	0.037

* Other includes COPD, Connective Tissue Diseases, Alpha1-antitrypsin deficiency, ABPA, Asthma, CFTR-RD, Aspiration. BSI: Bronchiectasis severity index; BACI: Bronchiectasis Aetiology and Co-Morbidity Index; FEV1: Forced expiratory volume first second.

## Data Availability

The data presented in this study are available on request from the corresponding author. The data are not publicly available due to privacy concerns.

## Supplementary Materials

The following are available online at www.mdpi.com/article/10.3390/biomedicines9111573/s1, Table S1: Clinical characteristics of the study population presented as median (IQR) or *n* (%), Table S2: SNPs in *GC* gene in bronchiectasis patients, Table S3: Comparison of clinical characteristics of patients with GC2 isoform vs. other isoforms, Table S4: Comparison of clinical characteristics of patients among isoform phenotypes.
