# Supplementary material for: The Isoform GC1f of the Vitamin D Binding Protein Is Associated with Bronchiectasis Severity
Source: Biomedicines. 2021 Oct 29;9(11):1573. doi: 10.3390/biomedicines9111573 (PMC8615139; doi:10.3390/biomedicines9111573)
Supplement: Supplementary file 1 [file biomedicines-09-01573-s001.zip › biomedicines-1399500-Supplementary material-update.pdf]

ONLINE SUPPLEMENT FOR

TITLE

The isoform GC1f of the vitamin D binding protein is associated to bronchiectasis severity

Authors

Martina Oriano [1,2,*], Stefano Aliberti [3,4,*], Franca Rosa Guerini [5,*], Cristina Agliardi [5], Carlotta Di Francesco [1,2], Alice Gelmini [1,2], Leonardo Terranova [1,2], Milena Zanzottera [5], Paola Marchisio [1,6], Mario Clerici [1,5,*] and Francesco Blasi [1,2]

Table S1. Clinical characteristics of the study population presented as median (IQR) or n (%)

**Demography**	
Sex (Female)	78 (67.2%)
Age	62.0 (48.8, 72.0)
BMI	21.7 (19.0, 24.9)
**Radiology**	
Reiff score	4.0 (3.0, 6.0)
**Disease severity**	
BSI score	6.0 (4.0, 9.0)
BSI risk classes	40 (34.5%)
	38 (32.8%)
	38 (32.8%)
FACED score	2.0 (1.0, 4.0)
FACED risk classes	63 (54.3%)
	41 (35.3%)
	12 (10.3%)
**Clinical status**	
Exacerbation in the previous year	2.0 (1.0, 3.0)
Hospitalization (at least one previous year)	13 (11.3%)
**Comorbidities**	
Osteoporosis	8 (6.9%)
Depression	12 (10.3%)
Anxiety	5 (4.3%)
Asthma	20 (17.2%)
**Lung function**	
FEV1%	84.0 (69.2, 99.0)
FEV1%<50%	15 (13.2%)
FEV1%<35%	8 (7.0%)
**Standard microbiology**	
Chronic infection	50 (49.0%)
Chronic *P. aeruginosa*	30 (29.4%)
Chronic *H. influenzae*	6 (5.9%)
Chronic *MSSA*	8 (7.8%)
Chronic *A. xylosoxidans*	2 (2.0%)

Chronic Others	4 (3.9%)
**Aetiology**	
Idiopathic	63 (54.3%)
Primary Ciliary Dyskinesia	10 (8.6%)
Primary immunodeficiency	13 (11.2%)
Post Infective	6 (5.2%)
Secondary Immunodeficiency	6 (5.2%)
Others*	
**QoL-B questionnaire**	
Physical section	60.0 (40.9, 80.0)
Role section	66.7 (53.3, 86.7)
Vitality section	55.6 (33.3, 69.5)
Emotion section	75.0 (58.3, 91.7)
Social section	66.7 (41.7, 83.3)
Treatment Burden section	66.7 (55.6, 77.8)
Health section	41.7 (22.2, 58.3)
Respiration section	74.1 (59.3, 81.5)
**Vitamin D**	
Vitamin D (ng/mL)	20.4 (13.0, 28.4)

* Other includes COPD, Connective Tissue Diseases, Alpha1-antitrypsin deficiency, ABPA, Asthma, CFTR-RD, Aspiration

Table S2: SNPs in *GC* gene in bronchiectasis patients

SNPs	Genotype	Bronchiectasis n (%)
rs7041	A A	21(18.1)%
	A C	58(50.0)%
	C C	37(31.9)%
rs4588	G G	61(52.6)%
	G T	47(40.5)%
	T T	8(6.9)%
**SNPs**	**Allele**	**Bronchiectasis n (%)**

rs7041	A	100 (43.1%)
	C	132(56.9%)
rs4588	G	169(72.8%)
	T	63(27.2%)

Table S3. Comparison of clinical characteristics of patients with GC2 isoform vs other isoforms

**Demography**				
		**GC2 (N=55)**	**Other isoforms (N=61)**	**p-value**
Sex (Female)		33 (60.0%)	45 (73.8%)	0.115
Age		62.0 (46.0, 71.0)	62.0 (54.0, 72.0)	0.453
BMI		22.0 (19.0, 25.8)	21.3 (19.0, 24.0)	0.328
**Radiology**				
Reiff score		4.0 (2.0, 5.0)	4.0 (3.0, 6.0)	0.77
**Disease severity**				
BSI score		5.0 (3.0, 9.0)	8.0 (4.0, 10.0)	0.081
BSI risk classes	mild	23 (41.8%)	17 (27.9%)	
	moderate	16 (29.1%)	22 (36.1%)	0.288
	severe	16 (29.1%)	22 (36.1%)	
BSI Moderate-severe		32 (58.2%)	44 (72.1%)	0.114
BSI Severe		16 (29.1%)	22 (36.1%)	0.424
FACED score		2.0 (1.0, 4.0)	2.0 (1.0, 4.0)	0.574
FACED risk classes	mild	31 (56.4%)	32 (52.5%)	
	moderate	18 (32.7%)	23 (37.7%)	0.854
	severe	6 (10.9%)	6 (9.8%)	
FACED Moderate-severe		24 (43.6%)	29 (47.5%)	0.673
FACED Severe		6 (10.9%)	6 (9.8%)	0.85
**Clinical status**				
Exacerbation in the previous year		2.0 (1.0, 3.0)	2.0 (1.0, 3.0)	0.397
Hospitalization (at least one previous year)		2 (3.6%)	11 (18.0%)	0.015
**Comorbidities**				
BACI		0.0 (0.0, 3.0	0.0 (0.0, 2.0)	0.317
Osteoporosis		1 (1.8%)	7 (11.5%)	0.040
Depression		4 (7.3%)	8 (13.1%)	0.302
Anxiety		1 (1.8%)	4 (6.6%)	0.209
Asthma		12 (21.8%)	8 (13.1%)	0.215
**Lung function**				
FEV1%		84.5 (70.0, 99.0)	81.5 (68.8, 101.0)	0.901
FEV1%<50%		7 (13.0%)	8 (13.3%)	0.953
FEV1%<35%		4 (7.4%)	4 (6.7%)	0.877
**Standard microbiology**				
Chronic infection		22 (50.0%)	28 (48.3%)	0.863
Chronic *P. aeruginosa*		15 (34.1%)	15 (25.9%)	0.366
Chronic *H. influenzae*		3 (6.8%)	3 (5.2%)	0.726
Chronic *MSSA*		2 (4.5%)	6 (10.3%)	0.281

Chronic *A. xylosoxidans*	2 (4.5%)	0 (0.0%)	0.101
Chronic Others	1 (2.3%)	3 (5.2%)	0.455
**Aetiology**			
Idiopathic	37 (67.3%)	26 (42.6%)	0.292
Primary Ciliary Dyskinesia	4 (7.3%)	6 (9.8%)	
Primary immunodeficiency	6 (10.9%)	7 (11.5%)	
Post Infective	2 (3.6%)	4 (6.6%)	
Secondary Immunodeficiency	2 (3.6%)	4 (6.6%)	
Others*	4 (7.3%)	14 (23.2%)	
**QoL-B questionnaire**			
Physical section	60.0 (46.7, 83.3)	63.4 (40.0, 80.0)	0.894
Role section	80.0 (53.3, 86.7)	66.7 (53.3, 86.7)	0.333
Vitality section	55.6 (33.3, 66.7)	55.6 (33.3, 72.2)	0.872
Emotion section	75.0 (50.0, 91.7)	79.2 (58.3, 91.7)	0.482
Social section	75.0 (41.7, 83.3)	57.0 (41.7, 83.3)	0.362
Treatment Burden section	66.7 (55.6, 77.8)	66.7 (55.6, 77.8)	0.913
Health section	41.7 (25.0, 66.7)	33.3 (20.8, 58.3)	0.627
Respiration section	74.1 (62.5, 81.5)	72.2 (58.3, 85.2)	0.919
**Vitamin D**			
Vitamin D (ng/mL)	19.5 (13.0, 26.8)	20.5 (13.2, 29.7)	0.536

* Other includes COPD, Connective Tissue Diseases, Alpha1-antitrypsin deficiency, ABPA, Asthma, CFTR-RD, Aspiration

Table S4. Comparison of clinical characteristics of patients among isoform phenotypes

**Demography**							
		**GC1s-GC1f (N.24)**	**GC1s-GC1s (N=37)**	**GC2-GC1f (N=13)**	**GC2-GC1s (N=34)**	**GC2-GC2 (N=8)**	**p-value**
Sex (Female)		17 (70.8%)	28 (75.7%)	9 (69.2%)	19 (55.9%)	5 (62.5%)	0.488
Age		61.0 (51.8, 72.2)	63.0 (54.0, 72.0)	62.0 (53.0, 69.0)	60.0 (44.5, 70.2)	66.0 (46.8, 72.8)	0.737
BMI		21.5 (19.7, 25.0)	21.3 (19.0, 24.0)	22.0 (19.0, 25.0)	21.9 (18.9, 25.4)	22.9 (21.0, 26.5)	0.739
**Radiology**							
Reiff score		4.0 (2.0, 6.0)	4.0 (3.0, 6.0)	4.0 (3.0, 6.0)	4.0 (2.0, 4.0)	3.0 (2.0, 4.2)	0.757
**Disease severity**							
BSI score		7.5 (3.8, 10.0)	8.0 (5.0, 9.0)	6.0 (5.0, 11.0)	5.0 (3.0, 8.8)	4.0 (3.8, 8.5)	0.196
BSI risk classes	mild	9 (37.5%)	8 (21.6%)	2 (15.4%)	16 (47.1%)	5 (62.5%)	0.184
	moderate	6 (25.0%)	16 (43.2%)	6 (46.2%)	9 (26.5%)	1 (12.5%)	
	severe	9 (37.5%)	13 (35.1%)	5 (38.5%)	9 (26.5%)	2 (25.0%)	
FACED score		2.0 (1.0, 3.2)	3.0 (1.0, 4.0)	3.0 (2.0, 4.0)	2.0 (1.0, 3.0)	2.5 (1.8, 4.0)	0.556
FACED risk classes	mild	15 (62.5%)	17 (45.9%)	6 (46.2%)	21 (61.8%)	4 (50.0%)	0.249
	moderate	5 (20.8%)	18 (48.6%)	4 (30.8%)	10 (29.4%)	4 (50.0%)	
	severe	4 (16.7%)	2 (5.4%)	3 (23.1%)	3 (8.8%)	0 (0.0%)	
**Clinical status**							
Exacerbation in the previous year		3.0 (2.0, 3.0)	2.0 (1.0, 4.0)	1.0 (1.0, 3.2)	2.5 (1.0, 3.0)	2.0 (1.8, 3.0)	0.878
Hospitalization (at least one previous year)		7 (29.2%)	4 (10.8%)	0 (0.0%)	0 (0.0%)	2 (25.0%)	0.005 (1)
**Comorbidities**							
BACI		0.0 (0.0, 2.2)	0.0 (0.0, 0.0)	0.0 (0.0, 3.0)	0.0 (0.0, 2.2)	3.0 (0.0, 4.2)	0.223

Osteoporosis	2 (8.3%)	5 (13.5%)	0 (0.0%)	1 (2.9%)	0 (0.0%)	0.289
Depression	2 (8.3%)	6 (16.2%)	0 (0.0%)	4 (11.8%)	0 (0.0%)	0.409
Anxiety	1 (4.2%)	3 (8.1%)	0 (0.0%)	1 (2.9%)	0 (0.0%)	0.663
Asthma	1 (4.2%)	7 (18.9%)	1 (7.7%)	7 (20.6%)	4 (50.0%)	0.039 (2)
**Lung function**						
FEV1%	81.0 (72.5, 102.5)	82.0 (68.0, 97.0)	73.0 (54.0, 99.0)	88.0 (72.0, 101.0)	84.0 (74.8, 86.5)	0.7
FEV1%<50%	3 (13.0%)	5 (13.5%)	3 (23.1%)	3 (9.1%)	1 (12.5%)	0.808
FEV1%<35%	1 (4.3%)	3 (8.1%)	2 (15.4%)	1 (3.0%)	1 (12.5%)	0.577
**Standard microbiology**						
Chronic infection	12 (57.1%)	16 (43.2%)	9 (75.0%)	13 (48.1%)	0 (0.0%)	0.059
Chronic *P. aeruginosa*	6 (28.6%)	9 (24.3%)	6 (50.0%)	9 (33.3%)	0 (0.0%)	0.267
Chronic *H. influenzae*	2 (9.5%)	1 (2.7%)	0 (0.0%)	3 (11.1%)	0 (0.0%)	0.467
Chronic *MSSA*	4 (19.0%)	2 (5.4%)	0 (0.0%)	2 (7.4%)	0 (0.0%)	0.248
Chronic *A. xylosoxidans*	0 (0.0%)	0 (0.0%)	1 (8.3%)	1 (3.7%)	0 (0.0%)	0.377
Chronic Others	1 (4.8%)	2 (5.4%)	1 (8.3%)	0 (0.0%)	0 (0.0%)	0.702
**Aetiology**						
Idiopathic	8 (33.3%)	18 (48.6%)	9 (69.2%)	21 (61.8%)	7 (87.5%)	0.631
Primary Ciliary Dyskinesia	5 (20.8%)	1 (2.7%)	0 (0.0%)	4 (11.8%)	0 (0.0%)	
Primary immunodeficiency	1 (4.2%)	6 (16.2%)	3 (23.1%)	2 (5.9%)	1 (12.5%)	
Post Infective	3 (12.5%)	1 (2.7%)	0 (0.0%)	2 (5.9%)	0 (0.0%)	
Secondary Immunodeficiency	3 (12.5%)	1 (2.7%)	1 (7.7%)	1 (2.9%)	0 (0.0%)	
Others*	4 (16.7%)	10 (27.1%)	0 (0%)	4 (11.7%)	0 (0%)	
**QoL-B questionnaire**						
Physical section	66.7 (43.4, 96.7)	60.0 (40.0, 74.2)	61.2 (46.6, 68.4)	63.4 (46.7, 85.9)	53.3 (30.0, 73.3)	0.72
Role section	73.3 (53.3, 93.3)	60.0 (48.4, 81.7)	70.0 (58.3, 88.4)	80.0 (55.0, 86.7)	80.0 (60.0, 86.7)	0.652
Vitality section	66.7 (47.2, 77.8)	55.6 (33.3, 66.7)	55.6 (44.4, 77.8)	55.6 (33.3, 66.7)	55.6 (38.9, 72.2)	0.803
Emotion section	83.3 (75.0, 95.8)	75.0 (54.1, 91.7)	91.7 (72.9, 100.0)	75.0 (50.0, 83.3)	58.3 (41.6, 66.7)	0.143
Social section	70.8 (33.3, 85.4)	55.6 (41.7, 72.9)	79.2 (60.5, 91.7)	66.7 (41.7, 83.3)	83.3 (70.8, 83.3)	0.654
Treatment Burden section	77.8 (63.9, 80.6)	55.6 (55.6, 77.8)	72.2 (58.4, 77.8)	66.7 (55.6, 77.8)	55.6 (50.0, 77.8)	0.535
Health section	44.4 (33.3, 66.7)	33.3 (16.7, 58.3)	38.8 (18.7, 68.8)	41.7 (27.1, 64.6)	50.0 (37.5, 50.0)	0.509
Respiration section	77.8 (66.7, 87.5)	66.7 (55.6, 82.4)	74.1 (65.8, 75.9)	75.9 (63.5, 81.5)	66.7 (63.0, 74.1)	0.643
**Vitamin D**						
Vitamin D (ng/mL)	20.5 (11.7, 27.9)	20.5 (14.8, 29.9)	13.4 (6.6, 24.6)	20.4 (15.9, 28.6)	17.8 (13.7, 21.0)	0.313

1) GC1s-GC2 vs GC2-GC2, p=0.003; GC1s-GC1f vs GC1s-GC2, p=0.001

(2) GC1s-GC1f vs GC2-GC2, p=0.002
